# Immune-Enhancing Effect of Submerged Culture of *Ceriporia lacerata* Mycelia on Cyclophosphamide-Induced Immunosuppressed Mice and the Underlying Mechanisms in Macrophages

**DOI:** 10.3390/ijms23020597

**Published:** 2022-01-06

**Authors:** Yong Pil Hwang, Gi Ho Lee, Thi Hoa Pham, Mi Yeon Kim, Chae Yeon Kim, Seung Yeon Lee, Eun Hee Han, Chul Yung Choi, Seong Deok Hwang, Sunyoung Ahn, Hye Gwang Jeong

**Affiliations:** 1 Fisheries Promotion Division, Mokpo 58613, Korea; protoplast@hanmail.net; 2 Department of Toxicology, College of Pharmacy, Chungnam National University, Daejeon 34134, Korea; ghk1900@cnu.ac.kr (G.H.L.); hoapt@cnu.ac.kr (T.H.P.); kmymy@o.cnu.ac.kr (M.Y.K.); chaeyeon05@o.cnu.ac.kr (C.Y.K.); sy9842@o.cnu.ac.kr (S.Y.L.); 3 Drug & Disease Target Research Team, Division of Bioconvergence Analysis, Korea Basic Science Institute (KBSI), Cheongju 28119, Korea; heh4285@kbsi.re.kr; 4 Department of Biomedical Science, College of Natural Science, Chosun University, Gwangju 61452, Korea; blockstar@chosun.ac.kr; 5 FugenCellTech Co., Ltd., Sangju-si 37272, Korea; sd.hwang@fugenbio.co.kr; 6 Bio-R&D Center, FugenBio Co., Ltd., Seoul 06740, Korea; sunyoung.ahn@fugenbio.com

**Keywords:** *Ceriporia lacerate*, immunomodulatory, cyclophosphamide, macrophages, cytokines, lymphocytes

## Abstract

The white-rot fungi *Ceriporia lacerata* is used in bioremediation, such as lignocellulose degradation, in nature. Submerged cultures and extracts of *C. lacerata* mycelia (CLM) have been reported to contain various active ingredients, including β-glucan and extracellular polysaccharides, and to exert anti-diabetogenic properties in mice and cell lines. However, the immunostimulatory effects have not yet been reported. This study aimed to identify the immunomodulatory effects, and underlying mechanisms thereof, of submerged cultures of CLM using RAW264.7 macrophages and cyclophosphamide (CTX)-induced immunosuppression in mice. Compared to CTX-induced immunosuppressed mice, the spleen and thymus indexes in mice orally administered CLM were significantly increased; body weight loss was alleviated; and natural killer (NK) cytotoxicity, lymphocyte proliferation, and cytokine (tumor necrosis factor [TNF]-α, interferon [IFN]-γ, and interleukin [IL]-2) production were elevated in the serum. In RAW264.7 macrophages, treatment with CLM induced phagocytic activity, increased the production of nitric oxide (NO), and promoted mRNA expression of the immunomodulatory cytokines TNF-α, IFN-γ, IL-1β, IL-6, IL-10, and IL-12. In addition, CLM increased the inducible NO synthase (iNOS) concentration in macrophages, similar to lipopolysaccharide (LPS) stimulation. Mechanistic studies showed that CLM induced the activation of the NF-κB, PI3k/Akt, ERK1/2, and JNK1/2 pathways. Moreover, the phosphorylation of NF-κB and IκB induced by CLM in RAW264.7 cells was suppressed by specific MAPKs and PI3K inhibitors. Further experiments with a TLR4 inhibitor demonstrated that the production of TNF-α, IL-1β, and IL-6 induced by CLM was decreased after TLR4 was blocked. Overall, CLM protected against CTX-induced adverse reactions by enhancing humoral and cellular immune functions, and has potential as an immunomodulatory agent.

## 1. Introduction

The mammalian immune system involves innate and adaptive immunity; good functioning of both is necessary to keep the body healthy [1]. The immune system is a sophisticated defense mechanism based on networks connected to the brain and endocrine system that react to external antigens and stimuli, and control the internal environment by removing abnormal cells and tissues from the body [2]. These immune systems are affected by various factors, resulting in increased or decreased efficacy of the immune system [3]. The study of materials that can alleviate disease by inhibiting or stimulating the immune response to antigens and stimuli is ongoing [4,5].

The loss of immune system homeostasis affects the immune response and leads to a variety of diseases, such as hypersensitivity diseases and immunosuppressive diseases [3,6]. Cyclophosphamide (CTX) is known to cause significant reductions in white blood cells (WBCs) by inducing severe hematopoietic and lymphatic damage when administered as an alkylating agent [7]. Therefore, it is widely recognized as a drug that inhibits the transplant rejection reaction; however, it also causes the same toxicity in cancer and normal cells (non-selective toxicity). Its side effects include anemia, hair loss, and a reduction in WBCs and platelets [8].

There is no clear optimal approach to immunity management. While many commercial drugs have been developed to control immunity, they are costly, and have harmful side effects and potentially serious complications such as autoimmunity, cytokine storm, and inflammation [9]. Therefore, the search for natural ingredients for immune treatment is important for the development of new therapeutics. Studies have shown that edible and medicinal mushrooms, such as *Phellinus baumii* and *Phellinus linteus*, have great therapeutic potential [10,11,12].

Recently, it has been confirmed that polysaccharides derived from natural products can enhance the immune system. Moreover, they have fewer side effects compared to synthetic drugs. Among the various polysaccharides, β-glucan is a natural polysaccharide produced by bacteria, yeast, fungi, and plants [5,13,14].

*Ceriporia lacerate*, first discovered in Japan in 2003 [15], is a type of white-rot fungi that decomposes cellulose and lignin in trees in the wild, playing a key role in biological reduction [16]. Cultured *C*. *lacerate* is composed of microscopic polypores and resembles a white moss. During liquid culture, various secondary metabolites, including extracellular polysaccharides (EPSs) and β-glucan, are produced; mycelial growth may also occur, depending on the environmental conditions.

FugenCelltech Co. Ltd. (Seoul, Korea) successfully developed a cultivation method for the green mass production of submerged culture medium of *C*. *lacerata* mycelia (CLM) and well-formulated antidiabetic nutraceutical tablets (Cepona^TM^). Submerged culture medium of CLM has been used to control high blood glucose levels [17,18] and insulin secretion via cytoprotective effects [19]. CLM also exhibits antihyperglycemic efficacy by lowering insulin resistance [20] and promotes insulin signal transduction via the activation of AMPK and GLUT4 [20,21]. Therefore, CLM is considered to have exceptional potential as a novel functional ingredient that can help control blood sugar in individuals with pre-diabetes by improving impaired glucose tolerance/insulin resistance. However, no studies have reported the immunostimulatory effects of CLM. In this study, we investigated the immune effects of submerged culture of CLM in mice with CTX-induced immunosuppression. In addition, the mechanism of action of the immune enhancement was studied in macrophages.

## 2. Results

### 2.1. Effect of CLM on Body Weight and Organ Indexes

**Figure 1:**
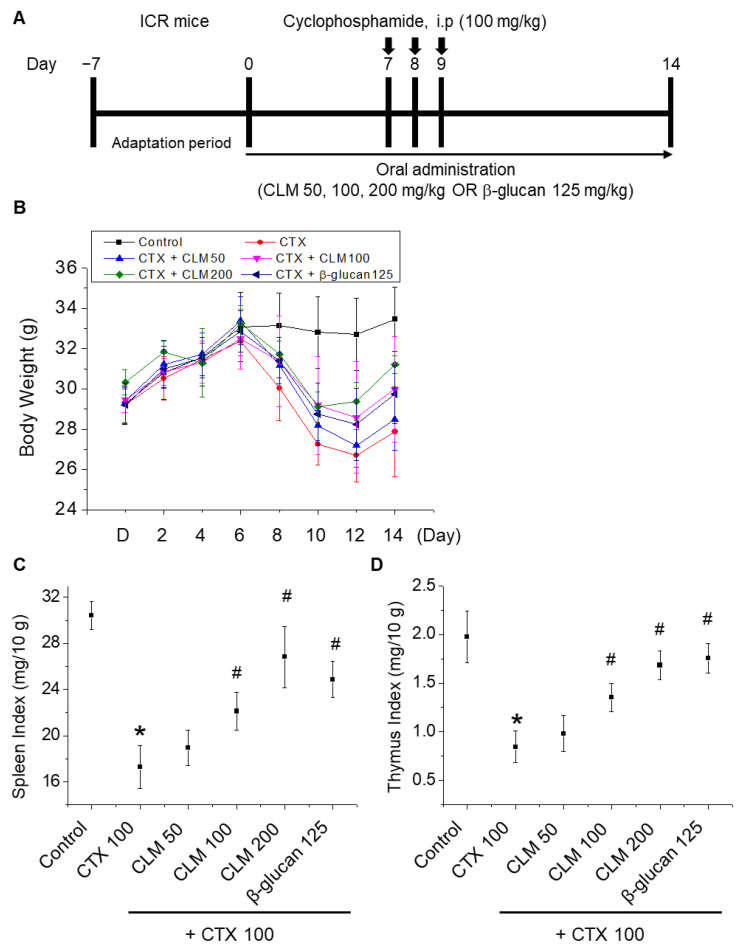

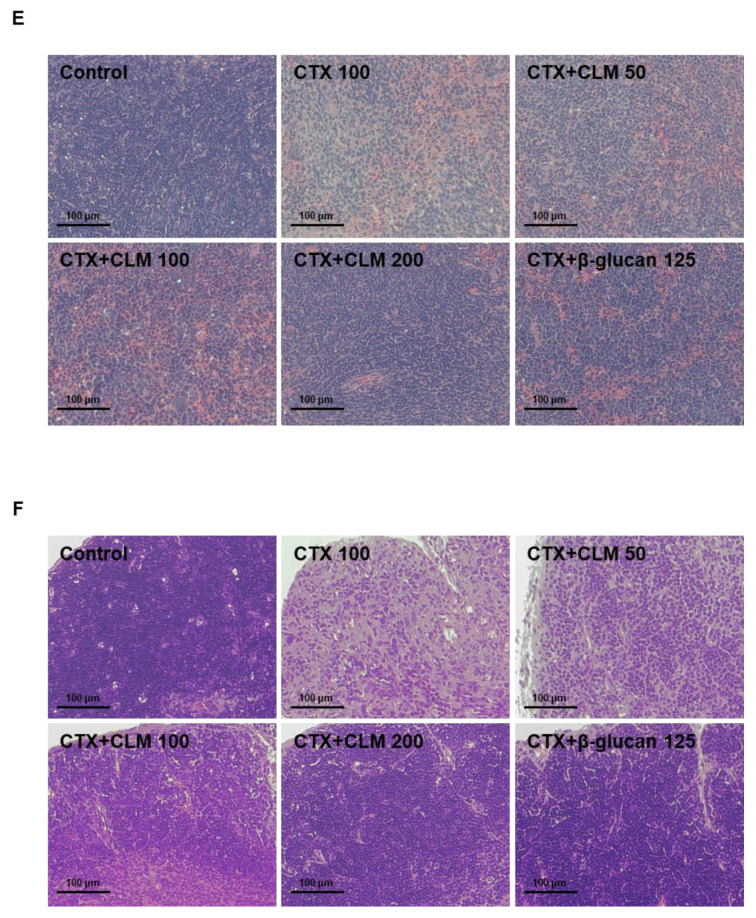
Influence of *C*. *lacerata* mycelia (CLM) on body weight, spleen index, and thymus index in cyclophosphamide (CTX)-treated mice. (**A**) General schematic diagram of the immunosuppression model and CLM treatment. Effect of orally administered CLM on body weight (**B**), spleen index (**C**), and thymus index (**D**) in CTX-immunosuppressed mice. CLM (50, 100, and 200 mg/kg/day) suspended in saline was administered orally once daily for 14 days. Mice were treated on days 7–9 by intraperitoneal injection of CTX (100 mg/kg/day) in a total volume of 100 µL of saline. The control group was treated with vehicle alone. The immune organ index (%) was calculated as follows: index = organ weight (mg)/body weight (g). The thymus and spleen were obtained on day 15 and weighed. Spleen (**E**) and thymus (**F**) tissues in the HE-stained histopathological images (total magnification 100×). Data are expressed as mean ± SD (*n* = 6). * *p* < 0.05 vs. normal group; # *p* < 0.05 vs. CTX-treated group.

Figure 1 shows the effect of CLM on body weight, and the spleen and thymus indexes of mice with CTX-induced immunosuppression. Compared to the vehicle-treated control, the body weight and spleen and thymus indexes of the CTX-treated group were reduced significantly. Following the administration of CLM, significant recovery in the body weight and the spleen and thymus indexes was seen compared to the CTX-treated group. The histomorphology of the spleen and thymus was examined with an optical microscope. The spleen (Figure 1E) and thymus (Figure 1F) of normal control mice had massive, closely arranged, and deeply stained splenocytes and thymocytes with an obvious nucleus. In the CTX-treated group, we observed a decrease in the number of splenocytes and thymocytes. In addition, there were necrotic areas with no cell structures in the HE-stained histopathological images. In contrast, in the CLM-treated groups, the spleen and thymus cells were arranged compactly, well ordered, with clear nuclei and less intercellular space, which was similar to the normal group. These results indicate that CLM significantly prevented the damage to the spleen and thymus in the mice induced by CTX, suggesting that CLM enhances the immune function of mice.

### 2.2. Effects of CLM on and Immunoglobulin (Ig) Levels, NK Cell Activity, and Lymphocyte Proliferation in CTX-Treated Mice

**Figure 2:**
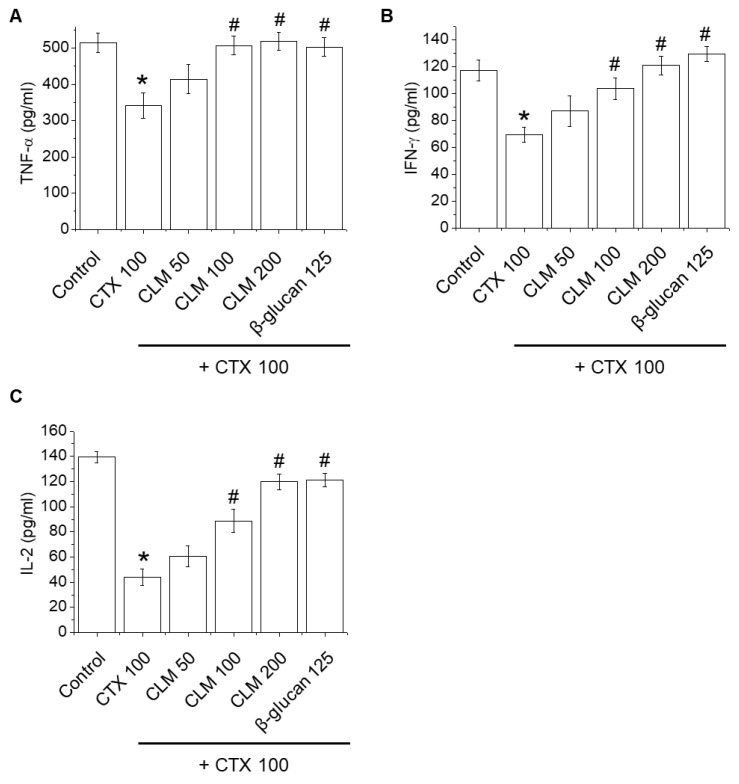
Effect of orally administered CLM on the levels of TNF-α (**A**), IFN-γ (**B**), and IL-2 (**C**) in CTX-immunosuppressed mice. CLM (50, 100, and 200 mg/kg/day) suspended in saline was administered orally once daily for 14 days. Mice were treated on days 7–9 by intraperitoneal injection of CTX (100 mg/kg/day) in a total volume of 100 µL of saline. The control group was treated with vehicle alone. Cytokines were assayed using commercial ELISA kits. Data are expressed as mean ± SD (*n* = 6). * *p* < 0.05 vs. normal group; # *p* < 0.05 vs. CTX-treated group.

**Figure 3:**
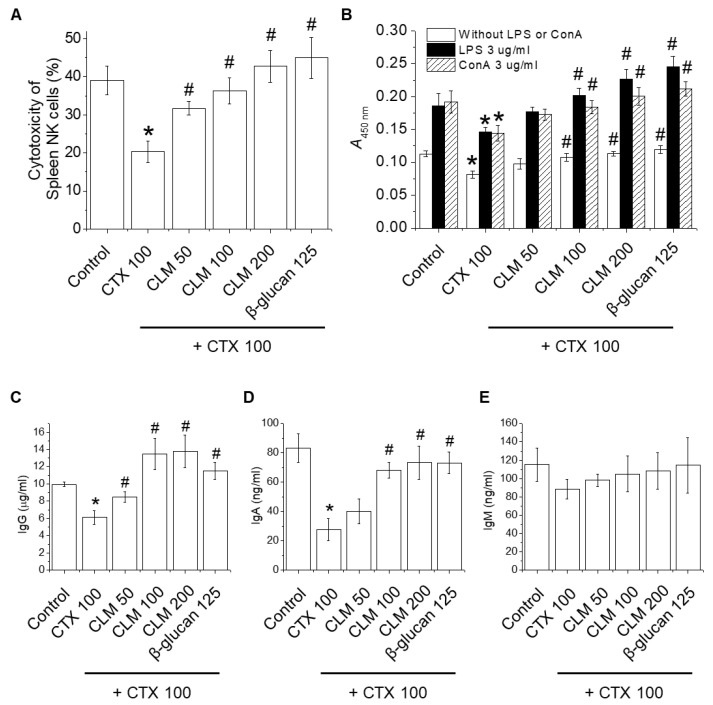
Effect of orally administered CLM on NK cell activity (**A**), LPS- or ConA-induced splenocytes proliferation (**B**), and immunoglobulin levels (**C**–**E**) in CTX-immunosuppressed mice. CTX-induced immunosuppressed ICR mice were treated with CLM (50, 100, and 200 mg/kg/day) for 14 days, and NK cell activity (**A**) and LPS- or ConA-induced splenocyte proliferation (**B**) were assessed. IgG (**C**), IgA (**D**), and IgM (**E**) were assayed using commercial ELISA kits. Data are expressed as mean ± SD (*n* = 6). * *p* < 0.05 vs. control group; # *p* < 0.05 vs. CTX-treated group.

Figure 2 and Figure 3 show the effects of CLM on cytokine and Ig levels, natural killer (NK) cell activity, and lymphocyte proliferation. In the CTX group, cytokine and Ig levels, NK cell activity, and lymphocyte proliferation were significantly decreased, indicating that CTX successfully induced immunosuppression. As shown in Figure 2, serum concentrations of tumor necrosis factor (TNF)-α, interferon (IFN)-γ, and interleukin (IL)-2 in CTX-treated mice were decreased significantly compared to the normal group. Treatment with CLM increased the concentrations of TNF-α, IFN-γ, and IL-2 compared to CTX-treated mice, similar to β-glucan. NK cell activity was significantly inhibited in the CTX-treated mice compared to the normal group (Figure 3A). Treatment with CLM significantly increased the NK cell activity in CTX-treated mice. The CLM group showed improved NK cell activity in a dose-dependent manner compared to the CTX group, similar to the β-glucan group. As shown in Figure 3B, the proliferation responses of spleen lymphocytes to T cell–concanavalin A (Con A) and B cell–lipopolysaccharide (LPS) stimuli were significantly inhibited in the CTX-treated mice compared with the normal group. CLM moderately elevated responses of both T and B lymphocytes in the CTX-treated mice. As shown in Figure 3C,D, production of IgG and IgA in the serum was significantly suppressed in CTX group compared to the normal group. CLM at 100 and 200 mg/kg significantly prevented the decline in IgG and IgA levels (Figure 3C,D), but CLM did not significantly regulate the IgM levels (Figure 3E).

### 2.3. Effects of CLM on Cell Viability and Macrophage Phagocytosis in Raw264.7 Cells

**Figure 4:**
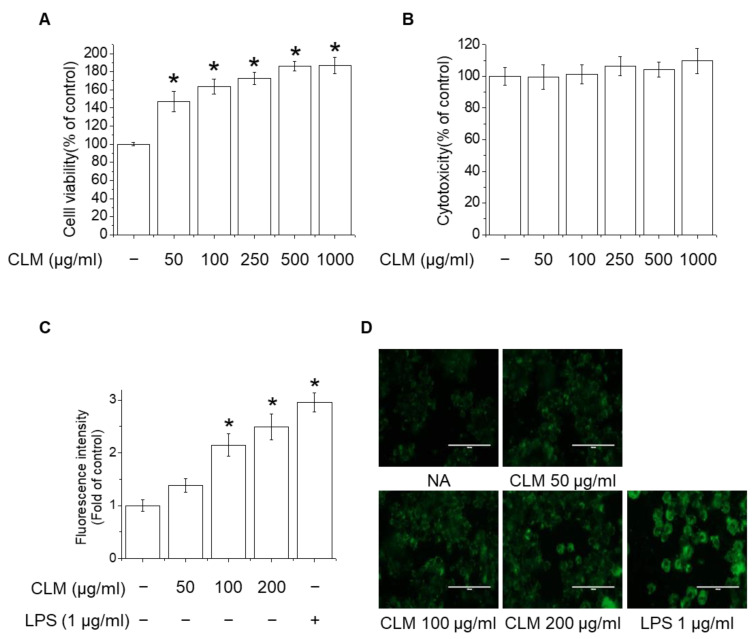
Effects of CLM on cell viability and phagocytic activity in macrophages. Cells were treated with various concentrations of CLM (50–1000 μg/mL) for 24 h. (**A**) Cell viability was determined by MTT assay. (**B**) Cell cytotoxicity was determined by LDH release assay. (**C**,**D**) Cells were treated with various concentrations of CLM (50, 100, and 200 μg/mL) for 24 h. After adding FITC-labeled *E*. *coli*, the cells were incubated for 2 h. (**C**) Cell culture medium containing unphagocytosed FITC-labeled *E*. *coli* was removed and fluorescence was measured with a microplate reader. (**D**) Fluorescence microscopy image of RAW264.7 cells stained with FITC-labeled *E*. *coli* for 2 h. All data are expressed as the mean ± SD of three independent experiments. * *p* < 0.01 vs. control group.

To determine the immunostimulatory effects of CLM, we first determined the cytotoxic effects of 50, 100, 250, 500, and 1000 μg/mL of CLM in RAW264.7 cells. The cytotoxicity and cell viability of CLM in RAW264.7 cells were evaluated using the MTT (Figure 4A) and lactate dehydrogenase (LDH) (Figure 4B) assays at 24 h. Concentrations of CLM up to 1 mg/mL did not affect cytotoxicity in RAW264.7 cells (Figure 4A,B). The immunostimulatory effects of CLM on macrophage phagocytosis were measured by internalization of FITC-labeled *Escherichia coli* particles using fluorescence microscopy (Figure 4C) and fluorescence spectrometry (Figure 4D). As shown in Figure 4C, the fluorescence intensity of the uptaken FITC-labeled *E*. *coli* was significantly enhanced in cells following treatment with CLM or lipopolysaccharide (LPS) for 24 h, compared to untreated RAW264.7 macrophages (Figure 4C,D).

### 2.4. Effects of CLM on Cytokines in RAW264.7 Macrophages

**Figure 5:**
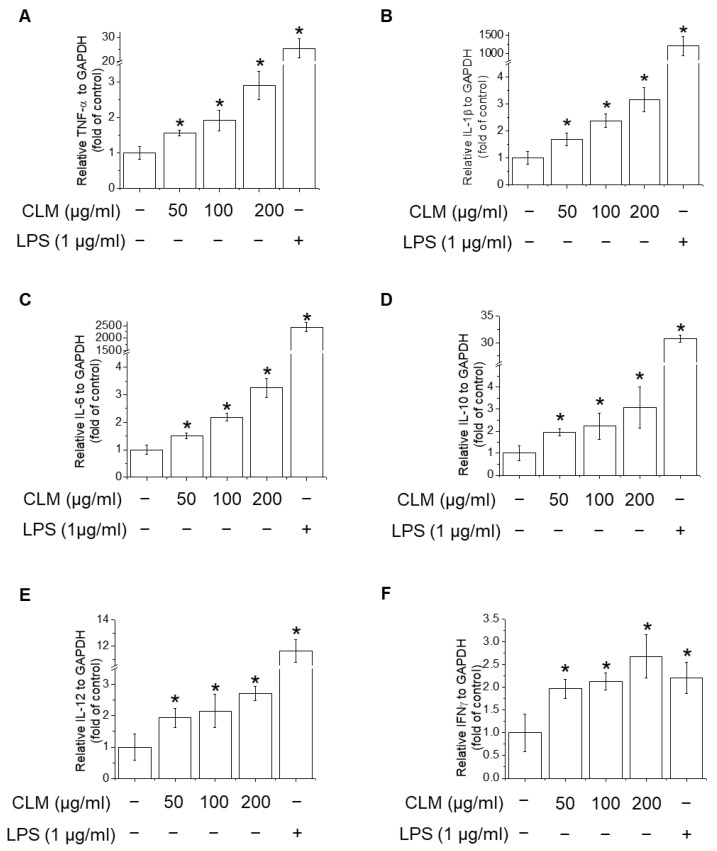
Effects of CLM on macrophage-related cytokine production in RAW264.7 cells. Cells were treated with various concentrations of CLM (50, 100, and 200 μg/mL) for 6 h. mRNA levels of TNF-α, (**A**), IL-1β (**B**), IL-6 (**C**), IL-10 (**D**), IL-12 (**E**), and IFN-γ (**F**) were measured by RT-PCR. * *p* < 0.01 vs. control group.

The effects of CLM on various inflammatory mediators of the immune system were studied and compared to the effects of LPS using RT-PCR. As shown in Figure 5, mRNA levels of TNF-α, IL-1β, IL-6, IL-10, IL-12, and IFN-γ were enhanced in CLM- and LPS-treated cells, suggesting that CLM is a potent inducer of cytokine secretion.

### 2.5. Effects of CLM on NO Production and iNOS Expression in RAW264.7 Macrophages

**Figure 6:**
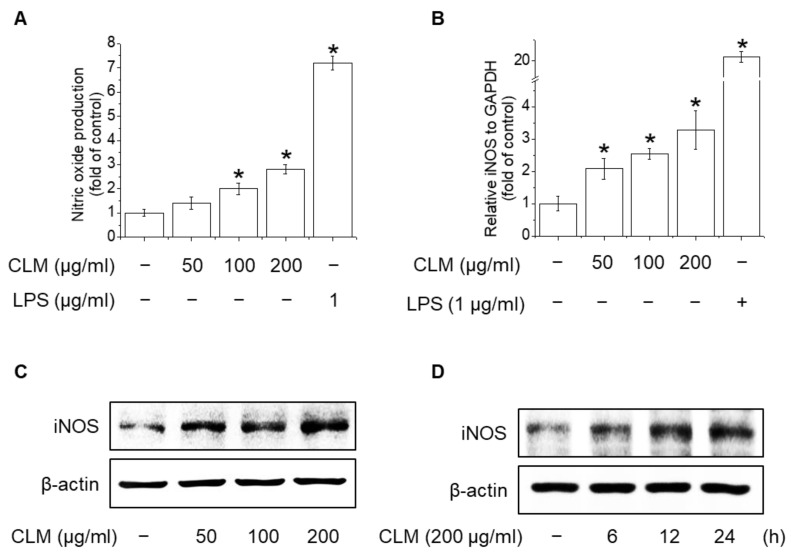
Effects of CLM on NO production and iNOS expression in macrophages. (**A**–**C**) RAW264.7 cells were seeded in a 12-well plate at a density of 2 × 10^5^ cells/mL for 24 h, cultured at 37 °C and 5% CO_2_, and treated with different concentrations of CLM (50, 100, and 200 μg/mL) and LPS (1 μg/mL) for 24 h. (**A**) Supernatants were harvested 24 h later and assayed for NO. (**B**) Total cellular RNA was extracted for RT-PCR with mouse iNOS primers. GAPDH confirmed the integrity and equal loading of RNA. (**C**) Cell lysates were electrophoresed and the expression of iNOS was detected with an iNOS antibody. (**D**) Cells were exposed to 200 μg/mL CLM and total cell lysate was extracted at the indicated times. The expression of iNOS was detected with an iNOS antibody; β-actin was measured as a loading control. * *p* < 0.01 vs. control group.

NO is a well-known signaling messenger in polysaccharide-stimulated immunomodulatory pathways. Several studies have suggested that NO possess antimicrobial activity and is positively correlated with the phagocytic activity of macrophages [22]. NO was measured as the concentration of nitrite produced by cells (Figure 6A). Inducible NO synthase (iNOS) is not always present in macrophages, but is influenced by various cytokines, such as TNF-α and LPS, which induce the expression of iNOS genes. The results suggest that NO production in macrophages is promoted by CLM and LPS (Figure 6A), and that CLM can improve the phagocytic capacity of macrophages. Therefore, iNOS expression was quantified by RT-PCR and western blotting. RAW264.7 cells treated with CLM or LPS showed significantly increased iNOS mRNA (Figure 6B) and protein expression (Figure 6C,D).

### 2.6. Effects of CLM on the Activation of NF-κB, MAPK, and PI3K/Akt Signaling Pathways in RAW264.7 Macrophages

**Figure 7:**
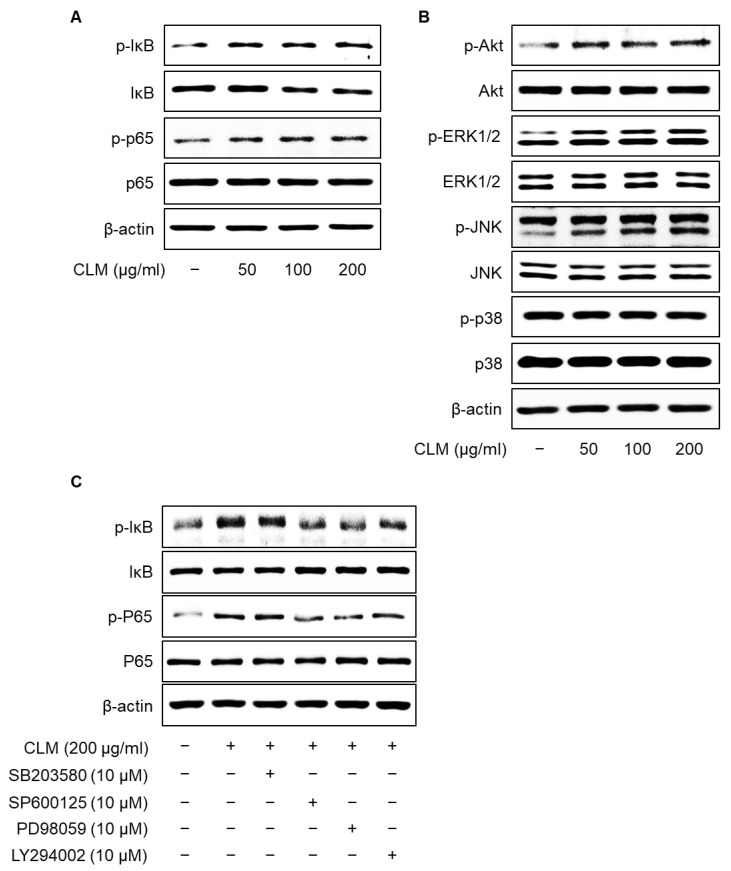
Effects of CLM-mediated NF-κB, MAPK, and PI3K/Akt activation in macrophages. (**A**,**B**) RAW264.7 cells were seeded in a 6-well plate at a density of 2 × 10^5^ cells/mL for 24 h, cultured at 37 °C and 5% CO_2_, and treated with CLM (50, 100, and 200 μg/mL) or LPS (1 μg/mL) for 1 h. The phosphorylation of IκB, NF-κB, Akt, ERK1/2, JNK1/2, and p38 was analyzed by western blotting. (**C**) Effects of PI3K and MAPK inhibitors on CLM-induced NF-κB activation. Cells were preincubated with 10 μM LY294002, 10 μM PD98059, 10 μM SB203580, or 10 μM SP600125 for 30 min, followed by incubation with 200 μg/mL CLM for 1 h. Cell lysates were subjected to western blotting with p-IκB, p-NF-κB, IκB, NF-κB, and β-actin antibodies; β-actin was used as the loading control.

NF-κB, MAPK, and PI3K/Akt can activate iNOS, facilitating initiation of the immunomodulatory response [23]. To investigate the molecular mechanism underlying CLM-mediated macrophage activation, activity in the NF-κB, MAPK (including ERK1/2, JNK1/2, and p38), and PI3K/Akt signaling pathways was detected by western blotting. We found that CLM could induce the phosphorylation of IκB, NF-κB, ERK1/2, JNK1/2, and Akt, but not p38, in RAW264.7 cells (Figure 7). Consistent with this, the signaling pathway inhibitors LY294002 (PI3K inhibitor), PD98059 (ERK inhibitor), and SP600125 (JNK inhibitor) inhibited the CLM-mediated activation of NF-κB and IκB, whereas SB203580 (p38 inhibitor) did not. The above data demonstrated that CLM could activate the MAPKs, PI3K, and NF-κB signaling pathways in RAW264.7 cells.

### 2.7. Effect of CLM on the TLR4-Mediated Cytokine Production in RAW264.7 Macrophages

**Figure 8:**
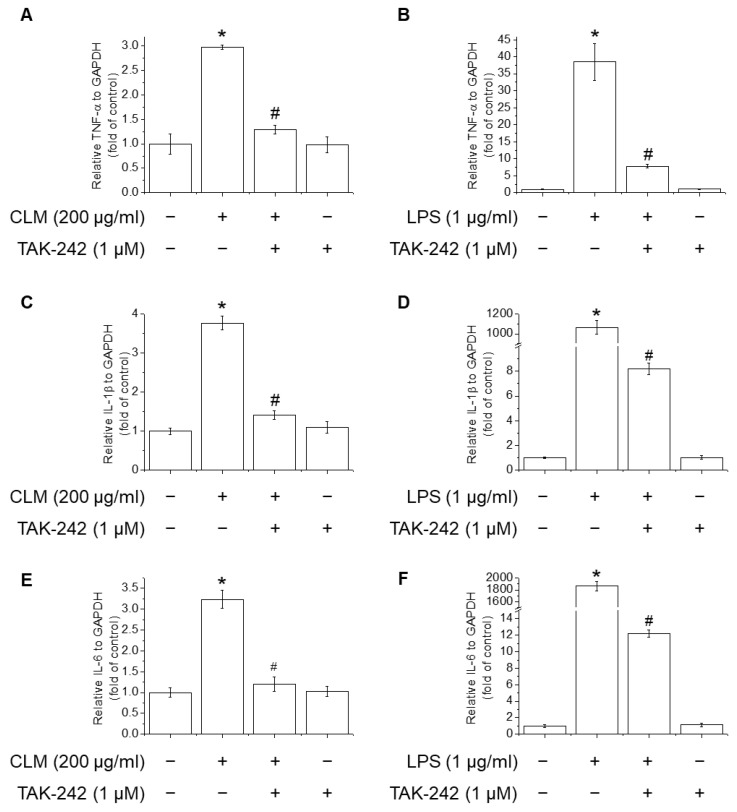
Effect of TLR4 inhibitor on CLM induced TNF-α, IL-1β, and IL-6 production in RAW264.7 cells. Cells were preincubated with 1 μM TAK-242 for 1 h, followed by incubation with 200 μg/mL CLM or 1 μg/mL LPS for 6 h. mRNA levels of TNF-α (**A**,**B**), IL-1β (**C**,**D**), and IL-6 (**E**,**F**) were measured by RT-PCR. * *p* < 0.01 vs. control group; # *p* < 0.01 vs. CLM-treated group.

In order to investigate the role of Toll-like receptors (TLR)4 played in the CLM-mediated stimulation of macrophages, TAK-242 (a TLR4 inhibitor) was pre-incubated with RAW264.7 cells for 1 h and then incubated with CLM or LPS. The production of TNF-α, IL-1β, and IL-6 in RAW264.7 cells was measured. As shown in Figure 8, the signaling pathway inhibitor TAK-242 inhibited the CLM-mediated expression of TNF-α, IL-1β, and IL-6. The above results showed that TLR4 could be a receptors of CLM.

## 3. Discussion

Immune function is essential to maintain health because it constitutes the defenses against infection. If immune function is lowered, the body becomes vulnerable to various infections; the effects of lowered immune function are an important clinical problem worldwide [24]. Various studies have been conducted to determine ways to improve immune function and identify natural materials without side effects [25]. Among the bioactive substances derived from natural products, β-glucans have been confirmed to effectively regulate immune function in the body and to defend against microbial and viral infections [26].

*Ceriporia lacerate*, a species of white-rot fungi that is saprophytic and lives on oak or pine trees, contains active ingredients such as EPSs and β-glucan. Only the use of submerged culture of CLM for diabetes treatment has been investigated to date [20,21]; the immunomodulatory effects have not yet been reported. In the present study, we investigated the immunomodulatory properties of CLM using macrophages and a mouse model of CTX-induced immunosuppression.

Natural product-derived bioactive functional foods and medicines have been reported to have lower toxicity than synthetic drugs, as well as less harmful effects on the human body, and to relieve inflammation, regenerate tissues, and prevent the recurrence of major cancers [27]. It is also known that natural product-derived polysaccharides promote the secretion of immune cells and cytokines to activate the immune system, and studies are underway to confirm the potential of various natural product-derived substances as immunomodulators [5,13,14].

CTX is commonly used as an antitumor drug with specific therapeutic effects on various tumors; it plays a role in immunosuppression and oxidative stress after hydrolysis, primarily via hepatocellular cytochrome P450 enzymes [7]. CTX has been reported to cause the atrophy of immune tissues, weight loss, and various imbalances in peripheral blood in mice, which can eventually inhibit immune function. Therefore, spleen and thymus morphology, and immune organ indexes were examined in this study. CTX markedly reduced the weight of the whole body, the spleen, and the thymus. In addition, CLM remarkably increased the weight of the whole body, the spleen, and the thymus in CTX-treated mice, and enhanced immune function. The administration of CLM increased the weight of the spleen and number of immune cells in the immunosuppression model, indicating that immune function was improved by CLM.

Lymphocytes are leukocyte subtypes in the immune system of vertebrates, and include NK, T and B cells; T cells are further classified as helper T (Th) cells, memory T cells, or NK T cells [28]. Th1 cells secrete IL-2 and IFN-γ, which are the main factors involved in cell-mediated immune responses. Th2 cells secrete IL-4, IL-6, and IL-10, which mediate immune responses [29]. IL-2 is produced by activated T cells and induces their growth, proliferation, and differentiation; IFN-γ promotes the differentiation of Th1 cells and the cellular immune response [30,31]. IL-2 and IFN-γ stimulate B cells to promote the production of Ig, leading to antibody immunoreactivity. Our results demonstrated that CLM increased the production of IL-2, IFN-γ, TNF-α, IgA, and IgG in CTX-induced immunosuppressed mice. In addition, CLM enhanced lymphocyte proliferative responses to T or B mitogen and NK cell activity in CTX-induced immunosuppressed mice. Thus, CLM can adjust immune imbalance and improve the symptoms of low immunity.

Macrophages are part of the innate immune system and play an important role in host defense mechanisms. Activated macrophages produce a variety of immunomodulators including proinflammatory cytokines and NO [32]. LPS is known to induce MAPK-dependent phosphorylation, thereby activating multiple transcription factors to translocate to the nucleus, resulting in the upregulation of TNF-α and iNOS expression [32]. In particular, NF-κB is a major activator of TNF-α production in macrophages and a major target for activators and inhibitors of iNOS expression [32]. Our results revealed the increased production of NO and the macrophage-related cytokines TNF-α, IFN-γ, IL-1β, IL-6, and IL-12 in mouse RAW264.7 macrophages, as well as enhanced iNOS expression. We also observed enhanced nuclear translocation of NF-κB.

Macrophage activation is regulated by the interaction of signal transduction systems in various cells. In particular, it has been demonstrated that the activation of the MAPK signaling pathway induces the activation of transcription factors such as NF-κB, thereby inducing the production of inflammatory mediators in macrophages [33]. Our results suggest that CLM directly stimulated macrophages by inducing the phosphorylation of Akt, ERK1/2, and JNK/12, but not p38. In addition, CLM-activated NF-κB was decreased by PI3K/Akt, ERK1/2, and JNK/12 inhibitors, indicating that CLM activates MAPK and PI3K signaling. According to recent reports, TLR4 is one of the most widely studied receptors for immune activity, mainly recognizing LPS, lipotechic acid, and plant polysaccharides [34]. Based on the results, we concluded that TLR4 was one of the membrane receptors of CLM in RAW246.7 cells. In this study, findings seem to indicate that CLM plays an immunostimulatory role in macrophages via the TLR4-induced production of TNF-α, IL-1β, and IL-6.

The present study demonstrated that the oral administration of CLM ameliorated CTX-suppressed cellular immunity by increasing the thymus and spleen indexes, activating T, B, and NK cells, and increasing the expression of immune modulators such as iNOS and cytokines. These findings suggest that CLM could function as an effective immunostimulatory agent in early innate immune responses. Further studies are required to identify the CLM-derived bioactive components responsible for immune enhancement.

## 4. Materials and Methods

### 4.1. Chemicals and Reagents

Roswell Park Memorial Institute (RPMI) 1640 medium, Dulbecco’s modified Eagle’s medium (DMEM), fetal bovine serum (FBS), streptomycin, and penicillin were obtained from Life Technologies (Carlsbad, CA, USA). Enzyme-linked immunosorbent assay (ELISA) kits for TNF-α, IFN-γ, and IL-2 were from R&D Systems (Minneapolis, MN, USA). 3-(4,5-Dimethylthiazol-2-yl)-2,5-diphenyltetrazolium bromide (MTT) was obtained from USB Corp. (Cleveland, OH, USA), and the LDH release detection kit was obtained from Roche Applied Science (Indianapolis, IN, USA). All kits were used according to the manufacturers’ protocols. CTX monohydrate, LPS, concanavalin A (ConA), and dimethylsulfoxide (DMSO) were obtained from Sigma-Aldrich (St. Louis, MO, USA). YAC-1 cells, a mouse lymphoma cell line, and RAW264.7 cells, a mouse macrophage cell line, were obtained from ATCC (Manassas, VA, USA; nos. ATCC^®^TIB-160^TM^ and ATCC^®^TIB-71 ^TM^). Antibodies against β-actin, iNOS, p-NF-κB p65, NF-κB p65, p-IκB, IκB, p-Akt, Akt, p-ERK1/2, ERK1/2, p-JNK1/2, JNK1/2, p-p38, and p38, and HRP-linked anti-rabbit IgG secondary antibody were obtained from Santa Cruz Biotechnology (Santa Cruz, CA, USA). All chemicals were of the highest grade commercially available.

### 4.2. Preparation of Submerged Culture C. lacerata Mycelia

The strain used in this study, originally owned by FugenCelltech, Co., Ltd. was cultured after inoculation with CLM in potato dextrose agar medium (Difco Co., Sparks, MD, USA) at 25 °C for 9 days. As a pre-culture process, the liquid medium of CLM was mixed with 4 g/L of starch, 20 g/L of glucose, and 600 mL of purified water for 10 days at pH 5, 23 °C, and 300 rpm, as previously described [16]. Following pre-culture, the mycelia culture was transferred to a liquid medium prepared by mixing 12.5 g/L of sugar, 2.5 g/L of skim soybean meal, 2.5 g/L of starch, 0.125 g/L of antifoam, and 400 L of purified water, and then adjusted to pH 5. The culture was incubated for 9 days at 23 °C with injection of air (1.0 kgf/cm^2^) at a rotational speed of 100 rpm. The complete culture of CLM was freeze-dried and pulverized, and used according to the capacity of each experimental group based on dry weight. This raw material was manufactured at a highly controlled GMP-certified plant at FugenCelltech Co., Ltd.

### 4.3. Preparation of the CTX-Induced Immunosuppression Mouse Model

Six-week-old male ICR mice (20 ± 2 g) were obtained from Samtako (Osan, Korea) and housed in a room with controlled temperature (22 ± 2 °C) and humidity (50 ± 5%), on a 12:12 h light/dark cycle with free access to food and water. The mice used in this study were handled in accordance with the Guidelines for the Care and Use of Laboratory Animals published by the US National Institutes of Health (NIH Publication NO. 85-23, 1996), and all experimental procedures were approved by the Committee on Ethics of Animal Experiments of Chungnam National University (202103A-CNU-066). After a 7 day acclimatization period, the mice were randomly divided into the following six groups (*n* = 6 per group): reference control (untreated), CTX control, CTX + β-glucan (125 mg/kg; positive control for preventive treatment), CTX + CLM50 (50 mg/kg), CTX + CLM100 (100 mg/kg), and CTX + CLM200 (200 mg/kg). Forty mice were injected intraperitoneally with CTX in sterile saline (100 mg/kg) for 3 consecutive days to establish immunosuppressed models (Figure 1A). CLM in saline was administered intragastrically at 50, 100, and 200 mg/kg, once daily for 14 days. Mice were treated on days 7–9 by intraperitoneal injection of CTX (100 mg/kg/day) in a total volume of 100 µL of saline. To measure body and spleen weights, mice were weighed on days 0 and 14. At the end of the experiment, mice were sacrificed by injection of 200 mg/kg pentobarbital, and organs, including the spleen, were immediately removed and weighed. The immune organ index (%) was calculated as follows: index = organ weight (mg)/body weight (g).

### 4.4. Isolation of Splenocytes

Spleen tissue was aseptically extracted from ICR mice (6-week-old males, 20 ± 2 g). Tissues were disaggregated via passage through a 70 μm nylon mesh (Becton-Dickinson, Franklin Lakes, NJ, USA) in RPMI-1640 medium (Life Technologies), and the cells were purified via centrifugation at 450× *g* for 5 min. Red blood cells (RBCs) were removed with ACK lysis buffer (Sigma-Aldrich). Splenocytes were then washed with phosphate-buffered saline (PBS), centrifuged at 1000× *g* for 5 min, and finally suspended in RPMI containing 10% FBS, penicillin, and streptomycin sulfate.

### 4.5. Assay of NK Cell Activity

Spleen tissue was aseptically extracted from each mouse, ground into a single-cell suspension using sterile gauze, and then washed three times with RPMI 1640 medium. Mouse spleen lymphocyte suspension was used as the effector cells. YAC-1 cells were used as the target cells. Briefly, the effector cells were centrifuged at 1000× *g* for 5 min at room temperature, and 100 μL spleen cell suspension (1 × 10^6^ cells/well) was seeded in a 96-well plate with the target cells (1 × 10^5^ cells/well) at an effector cell:target cell ratio of 10:1; 100 μL RPMI 1640 medium was used as a control. After 4 h at 37 °C and 5% CO_2_, the plate was centrifuged at 800× *g* for 5 min, the culture supernatant (100 μL/well) was mixed with LDH solution (Promega, Madison, WI, USA), and the absorbance of each well was measured at 490 nm. NK cell cytotoxicity was calculated as follows: cytotoxicity (%) = [(experimental release − spontaneous release)/(maximum release − spontaneous release)] × 100.

### 4.6. Assay of Splenocyte Proliferation

Splenocyte proliferation was examined using water-soluble tetrazolium (WST)-1 assay kit (Roche Diagnostics, Mannheim, Germany). Splenocytes were seeded in 96-well plates at density of 1 × 10^5^ cells/well in DMEM with 10% FBS. Cells were respectively treated with RPMI 1640 medium (without LPS or Con A) or LPS (3 μg/mL) or Con A (3 μg/mL) for 48 h, and then 10 μL of WST-1 reagent for 2 h was added. The absorbance of each well was measured at 450 nm with a microplate reader (Varioskan; Thermo Fisher Scientific, Waltham, MA, USA).

### 4.7. Measurement of Serum Cytokine and Ig Levels

Cytokine and Ig levels in blood samples were quantified using ELISA kits according to the manufacturer’s instructions. Briefly, the blood of mice was taken from the eyeballs, left to stand for 30 min, and centrifuged at 3500 r/min for 10 min at 4 °C. After the serum was taken, the levels of TNF-α, IFN-γ, IL-2, IgM, IgA, and IgG by ELISA kit were determined.

### 4.8. Cell Culture and Sample Treatment

Mouse splenocytes, YAC-1 cells, and RAW 264.7 macrophages were cultured in RPMI 1640 and DMEM medium containing 10% FBS, streptomycin sulfate, and penicillin at 37 °C and 5% CO_2_. Cells were treated with various concentrations (50, 100, 200 μg/mL) of CLM, LPS (3 μg/mL), and Con A (3 μg/mL).

### 4.9. Measurement of Cell Cytotoxicity

Cell viability and cytotoxicity was assessed with the MTT or LDH assay. RAW264.7 mouse macrophages (1 × 10^6^ cells/well) were seeded in 96-well plates containing 100 μL RPMI 1640 medium and 10% FBS, and incubated for 24 h. CLM (50–1000 μg/mL) was added, followed by incubation for 24 h. MTT solution (5 mg/mL in PBS) was added for 30 min at 37 °C and 5% CO_2_; the medium was then discarded, formazan crystals were dissolved with 100 μL DMSO, and the absorbance of each well at 550 nm was measured with a microplate reader. The medium was collected for LDH assay and then mixed with 50 μL LDH solution. The absorbance at 490 nm was measured using a microplate reader. Calculations of cell viability (%) and cytotoxicity (%) were based on the absorbance of treated cells relative to that of cells exposed to DMSO alone.

### 4.10. Assay for Macrophage Phagocytosis

The phagocytosis assay was performed as previously described [35]. Peritoneal macrophages of ICR mice were aseptically harvested by peritoneal lavage with 5 mL PBS. The cells were seeded in 96-well plate with a complete RPMI-1640 medium at a density of 1 × 10^6^ cells/well and cultured at 37 °C in 5% CO2 for 24 h and then, 100 μL fluorescein-5-isothiocyanate (FITC)-labeled *Escherichia coli* (Molecular Probes, Eugene, OR, USA) was added for various times. Extracellular fluorescence was quenched by adding 100 mL of Trypan blue. After 1 min, FITC-labeled bacteria that had not been phagocytosed by macrophages were washed away, and the macrophages were rinsed twice with PBS and lysed with lysis buffer (10 mM Tris–HCl [pH 7.5], 130 mM NaCl, 1% Triton X-100, 10 mM Na_2_HPO_4_, and 10 mM Na_4_P_2_O_7_). The relative fluorescence intensity of bacteria inside the macrophages was determined at excitation and emission wavelengths of 480 and 520 nm, respectively, using a microplate reader. The relative phagocytic activity was calculated as the percentage fluorescence intensity of sample-supplemented versus non-supplemented (control) FITC-labeled bacteria.

### 4.11. Nitrite Assay

RAW 264.7 cells (5 × 10^5^ cells/well) were cultured in 96-well plates and stimulated with or without various concentrations of CLM (50, 100, and 200 μg/mL). After incubation for 24 h, the culture supernatant was collected, and NO level was measured by using Griess reagent assay. Equal volumes of Griess reagent (1:1 of 1% sulfanilamide in 5% phosphoric acid and 0.1% N-1 naphthylethylenediamine in 5% phosphoric acid) and sample were incubated together at room temperature for 5 min. Absorbance at 550 nm was measured using a microplate reader (Varioskan; Thermo Fisher Scientific).

### 4.12. Western Blotting

RAW264.7 macrophages were treated with LPS (1 μg/mL) or CLM (50, 100, and 200 μg/mL) for 24 or 0.5 h, and protein levels of iNOS, p-NF-κB, NF-κB, p-IκB, IκB, p-Akt, Akt, p-ERK1/2, ERK1/2, p-JNK1/2, JNK1/2, p-p38, and p38 were determined by immunochemistry. Total and nuclear protein concentrations of the supernatants were estimated using the Bradford method; 50 μg of protein was separated by 10% SDS-PAGE and transferred onto a polyvinylidene difluoride membrane. RAW264.7 macrophages were cultured with CLM. Total cellular protein (50 μg) was resolved by 10% SDS-PAGE and transferred onto a polyvinylidene difluoride membrane. After blocking, the membranes were incubated with the target antibody, followed by incubation with a horseradish peroxidase-conjugated secondary antibody against IgG. The blots were probed using the ECL Western blot detection system, as instructed by the manufacturer (BioFact, Daejeon, Korea).

### 4.13. Real-Time PCR

Total RNA was extracted from RAW264.7 cells using the RNAiso Plus kit. Total RNA extraction reagent (Takara, Shiga, Japan) was used and cDNA was synthesized using the BioFact RT Series kit. PCR amplification was monitored by CFX Connect Real-Time PCR software (version 1.4.1; Bio-Rad Laboratories, Hercules, CA, USA). The primers used are listed in Table 1.

### 4.14. Statistical Analysis

Data are shown as mean ± standard deviation (SD) of triplicate experiments. Data from the animal study are expressed as mean ± SD (*n* = 6). Mean differences between treatments groups were evaluated using one-way analysis of variance (ANOVA) by Dunnett’s post hoc test and *p* values < 0.05 or < 0.01 were considered statistically significant.

## 5. Conclusions

The present study demonstrated that the submerged culture and extracts of *C. lacerata* mycelia (CLM) could alleviate cyclophosphamide-induced immunosuppression in mice by protecting the spleen and the thymus, increasing NK cell activity and splenocytes proliferation, and restoring the levels of TNF-α, IFN-γ, IL-2, IgA, and IgG in the serum. The in vitro study showed that CLM had an immunostimulatory effect on RAW264.7 cells, as evidenced by the increased proliferation and cell cycle, enhanced phagocytosis ability, and upregulated production of NO and TNF-α, IL-1β, IL-6, IL-10, IL-12, and IFN-γ cytokines. In addition, our results show that the PI3K/Akt, ERK1/2, JNK/12, and TLR4 signaling pathways are responsible for these effects.

## Figures and Tables

**Table 1:** Primer sequences for real-time PCR.

Gene		Sequences
Mouse GAPDH	F	AAAAGGGTCATCATCTCCGC
	R	ATTTCTCGTGGTTCACACCC
Mouse TNF-α	F	CGGGCAGGTCTACTTTGGAG
	R	ACCCTGAGCCATAATCCCCT
Mouse IFN-γ	F	GAGGTCAACAACCCACAGGT
	R	GGGACAATCTCTTCCCCACC
Mouse IL-1β	F	GAAGGGCTGCTTCCAAACCT
	R	TGATGTGCTGCTGCGAGATT
Mouse IL-6	F	TACCACTTCACAAGTCGGAGG
	R	CTGCAAGTGCATCATCGTTGTT
Mouse IL-10	F	GCCCTTTGCTATGGTGTCCTT
	R	GGCCACAGTTTTCAGGGATGA
Mouse IL-12	F	CGCCACACAAATGGATGCAA
	R	TGTGTCCTGAGGTAGCCGTA
Mouse iNOS	F	GCATCCCAAGTACGAGTGGT
	R	GGTGCCCATGTACCAACCAT

## Data Availability

The data presented in this study are available on request from the corresponding author.
